# *Emiliania huxleyi* coccolith calcite mass modulation by morphological changes and ecology in the Mediterranean Sea

**DOI:** 10.1371/journal.pone.0201161

**Published:** 2018-07-24

**Authors:** Barbara D’Amario, Patrizia Ziveri, Michaël Grelaud, Angela Oviedo

**Affiliations:** 1 Institute of Environmental Science and Technology (ICTA), Universitat Autònoma de Barcelona (UAB), Bellaterra, Spain; 2 Institució Catalana de Recerca i Estudis Avançats (ICREA), Barcelona, Spain; Fred Hutchinson Cancer Research Center, UNITED STATES

## Abstract

To understand the response of marine calcifying organisms under high CO_2_ scenarios, it is critical to study their calcification patterns in the natural environment. This paper focuses on a major calcifying phytoplankton group, the coccolithophores, through the analysis of water samples collected along a W-E Mediterranean transect during two research cruises, in April 2011 (Meteor cruise M84/3) and May 2013 (MedSeA cruise 2013). The Mediterranean Sea is a marginal sea characterized by large biogeochemical gradients. Currently, it is undergoing both warming and ocean acidification, processes which are rapidly modifying species distribution and calcification. The species *Emiliania huxleyi* largely dominates the total coccolithophore production in present day oceans and marine basins, including the Mediterranean Sea. A series of morphometric measurements were performed on the coccoliths of this species to estimate their mass, length and calculate a calcification index (proxy for the size-normalized calcification degree). The most abundant morphotype of *E*. *huxleyi* in the Mediterranean Sea is Type A. Coccoliths of this morphotype were additionally analyzed based on scanning electron microscopy images: four calcification varieties were quantified, according to the relationship between slit length-tube width, and the state of the central area (open or closed). The average *E*. *huxleyi* coccolith mass along the Mediterranean oceanographic transect depended strongly on both the average coccolith length and calcification index. The variability in average coccolith length and calcification index across samples reflected oscillations in the relative abundance of the calcification varieties. We also demonstrated that the distribution of the calcification varieties followed the main environmental gradients (carbonate chemistry, salinity, temperature, nutrient concentrations). Hence, shifts in the distribution of the calcification varieties and of the average *E*. *huxleyi* coccolith mass are to be expected in the Mediterranean Sea under climate change. These physiological and ecological responses will modulate the net coccolithophore calcification and, ultimately, the regional carbonate export to the seafloor.

## 1 Introduction

The accumulation of human-induced atmospheric CO_2_ is altering the global climate and driving rapid changes in the carbonate chemistry of surface seawaters. For example, it has been estimated that since the industrial revolution, the global ocean surface pH has decreased by 0.1 units and a supplementary decrease of 0.06 to 0.32 pH units is expected by the end of the 21^st^ century [1,2]. This process, termed ocean acidification, is thought to impact the calcification process of many marine organisms [3,4]. However, it is still unknown how large such potential reduction in calcification would be, or what could be the net effect on coccolithophores, which represent the most prominent calcifying phytoplankton group on Earth: their calcite builds up 20 to 40% of the total open ocean carbonate sedimentation, from equatorial to sub-polar regions [5–7].

Several studies have shown the sensitivity to ocean acidification of the family Noelaerhabdaceae, the most abundant in present-day coccolithophore communities [8–12]. On a global scale, coccolith mass within this family seems correlated to the seawater carbonate system (carbonate ion concentration, calcite saturation state, pH, *p*CO_2_): carbonate chemistry could control the distribution of differently calcified taxa, primarily species and morphotypes of the genera *Gephyrocapsa* and *Emiliania* [12–14]. Most laboratory experimental results on selected strains of *Emiliania huxleyi*, as reviewed by [8], showed a tendency for decreasing PIC production and PIC: POC ratio in high *p*CO_2_ conditions. Nonetheless, variable and sometimes contradictory responses have been found with respect to ocean acidification for *E*. *huxleyi*, e.g. [12,15,16]. This is probably due to the large number of genotypes included in the species considered [17–20]. Also, in the natural environment, the regional distribution of differently calcified specimens of *E*. *huxleyi* is likely affected by additional parameters [21], such as temperature [22], nutrient concentrations [23] and salinity [24].

Overall, the responses of *E*. *huxleyi* to changing environmental conditions have been examined through two main approaches: field studies and laboratory experiments. Field studies describe natural coccolithophore communities and their geographical distributions: they can highlight statistically significant correlations between the distribution of morphotypes, calcification varieties and the environmental variables, but cannot be used to establish cause-effect relationships. Conversely, laboratory experiments can prove the direct effects of different environmental pressures on *E*. *huxleyi*, but only for a few strains and environmental parameters at a time: they cannot replicate the complexity of the natural environment, and as such they cannot account for genotype sorting.

*Emiliania huxleyi* was initially classified into four genotypically-controlled morphotypes: A, B, C and corona [25]. The combined approach of microscopic and molecular techniques has allowed the distinction of other morphotypes, highlighting their genetic diversity [17,18,26–31] and biogeographical/seasonal distributions [18,32–34]. Up to present, five morphotypes of *E*. *huxleyi* (A, B, B/C, C and R) are proven to remain consistent when reproducing [25,35,36].

The existence of different degrees of calcification within *E*. *huxleyi* Type A has been documented in the past [35], mainly from the North Atlantic Ocean [37–39], the Pacific Ocean [40] and the Mediterranean Sea [41–47]. The region of focus for this study is the Mediterranean Sea: a ‘small-scale ocean’ with steep W−E biogeochemical and physical gradients and a fast overturning circulation (80 to 100 years) [48].

*E*. *huxleyi* calcification (coccolith mass) in the Mediterranean Sea is controlled by the average coccolith size and calcification degree. The present work hypothesizes that such morphological parameters are linked to different proportions of Type A calcification varieties vs. Type B/C. Four Type A calcification varieties are described here and their spatial distribution is evaluated to identify plausible environmental controls.

## 2 Material and methods

### 2.1 Environmental conditions

**Fig 1:**
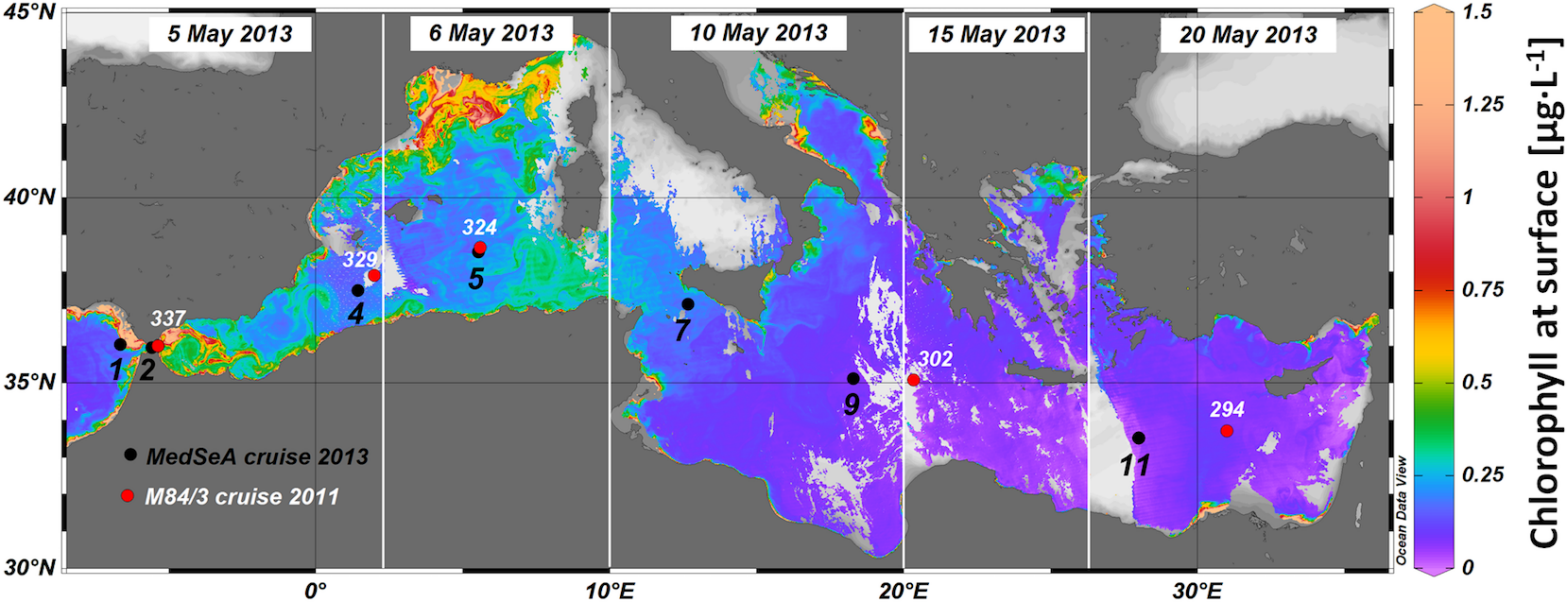
Location of sampled stations and superficial Chl *a* during the MedSeA cruise. The superficial Chl *a* was obtained from satellite data (MODIS Aqua L2).

The oceanographic data presented and discussed in this paper were retrieved along a W-E transect extending from the Atlantic Ocean (-6.64° E, 36.03° N) to the Levantine Basin of the Mediterranean Sea (31.00° E, 33.70° N; Fig 1), during the Meteor M84/3 (6 − 28 April 2011) and the MedSeA (1 − 31 May 2013) oceanographic cruises, following similar longitudinal transects (S1 Fig) [49,50]. Three main water masses were identified within the upper 200 m during the MedSeA and M84/3 cruises, as shown in the temperature-salinity diagram (S2 Fig). Detailed descriptions of the oceanographic settings and the methods of analysis used have been published previously for both the Meteor M84/3 cruise [49,51,52] and the MedSeA cruise [50]. Permission for navigation and research operations in exclusive economic zones of the Mediterranean Sea was granted from the Governments of Spain, Greece, France, Italy and Cyprus. Sampling did not involve endangered or protected species.

### 2.2 Phytoplankton samples

A total of 55 water samples from the two cruises were analyzed through morphometric measurements (mass, length, width) and/or detailed morphology recognition. They were collected along the transect (Fig 1) between 5 and 150 m depth (Table 1), following the eastward deepening of the photic zone. The depth of the photic zone was estimated from the available fluorescence values (see fluorescence profile in the supplementary material of [50]). Using Niskin bottles, 2.5–5 liters of water per sample were collected and filtered through cellulose acetate-nitrate filters (Millipore, Ø 47 mm, 0.45 μm). A hydraulic vacuum pump system (Eyela, A-1000S) was used at low pressure, to obtain an even distribution of particles on the filter. Each filter was then rinsed with distilled water, buffered with ammonia (63 ml NH_3_ + 500 ml of distilled water), in order to remove salt residues, and oven-dried at 40°C for 8–12 hours.

**Table 1:** List of samples analyzed using SYRACO (a) and/or SEM (b).

Station	Date	Coordinates		Depth (m)										
	dd/mm/yy	°E	°N	5	10	25	40	50	75	80	100	110	125	150
1	02/05/13	-6.64	36.03	a,b		a	a,b							
2	03/05/13	-5.56	35.95	a,b	a,b	a		a,b	a					
4	07/05/13	1.45	37.49	a,b		a	a,b				a			
5	08/05/13	5.55	38.52	a		a,b		a	a	a,b			a	
7	11/05/13	12.68	37.12	a,b		a		a			a,b			
9	12/05/13	18.29	35.11	a		a,b		a				a	a	a,b
11	15/05/13	28.00	33.50	a		a,b		a	a		a,b		a,b	a
294	10/04/11	31.00	33.70	a,b		a,b		a,b			a,b			
302	13/04/11	20.35	35.07			a,b		a,b			a,b			
324	21/04/11	5.60	38.65	a,b		a,b		a,b			a,b			
329	23/04/11	2.00	37.90	a,b		a,b		a,b			a,b			
337	25/04/11	-5.36	36.00	a,b		a,b		a,b			a,b			

#### 2.2.1 *Emiliania huxleyi* coccolith mass and length

For each sample, a portion of filter was mounted on a glass slide, soaked in immersion oil and secured applying a coverslip and tape on the borders of the slide. The sample slides were then analyzed with a Leica DM6000B cross polarized light microscope fitted to a SPOT Insight Camera, at x 1000 magnification. The luminosity level of the microscope was set prior to analysis, following the guidelines of [21]. At least 40 pictures were taken per sample, to count a minimum of 300 intact and isolated coccoliths (100 in a few low abundance samples). The resulting images were analyzed with an automated system of coccolith recognition (SYRACO, [53,54]). The mass (pg) and length (μm) of 23255 individual isolated coccoliths were measured following the guidelines of [21,55]. The input of reworked calcareous nannofossils from the continent is a common process in the Mediterranean Sea, as shown by previous studies on Holocene sediments (i.e. [56–60]). However, scanning electron microscopy (SEM) observations on the same samples did not reveal any evident sign of diagenesis on the specimens. Because of this, we considered the analyzed coccoliths to be a representation of those originally interlocked in *E*. *huxleyi* coccospheres.

#### 2.2.2 *Emiliania huxleyi* morphotypes and Type A calcification varieties

A portion of filter was radially cut from each sample, attached to a stub and coated with a gold/palladium (Au/Pd) alloy, to be observed at 30000 X magnification through SEM (Zeiss EVO MA 10). A longitudinal transect of 5 mm or more was scanned (≈ 4.1 ml of water), until a minimum of 80 − 100 coccospheres were counted: *Emiliania huxleyi* morphotypes were identified and their coccospheres counted separately.

**Fig 2:**
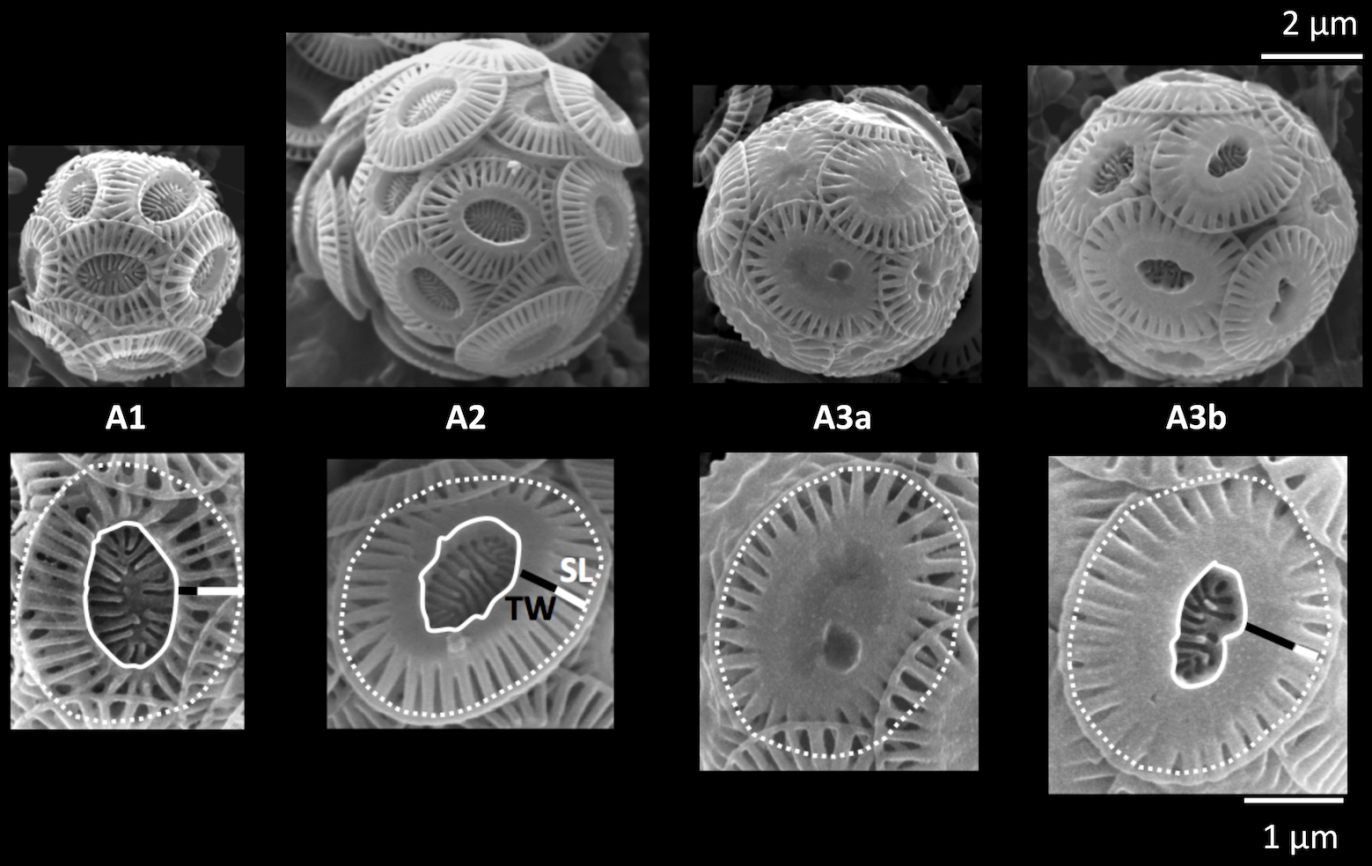
Calcification varieties of *Emiliania huxleyi* Type A. The open central area is highlighted with a curved continuous line; the outer limit of the slits by a curved dotted line. TW = tube width (black bar), SL = slit length (white bar). The scale of 2 μm refers to the coccospheres, while the scale of 1 μm to the coccoliths.

Individual *E*. *huxleyi* coccoliths from Type A coccospheres (a mean of 377 per sample, for a total of 13192) were identified by SEM observations and assigned to one of the following categories: low−calcified (A1), medium−calcified (A2) and high−calcified (A3a, A3b). The main distinguishing feature between A1, A2 and A3b was the ratio between slit length (SL) and tube width (TW) on the distal shield of the coccolith (Fig 2): in A1, SL > TW; in A2, SL ≈ TW; in A3b, SL < TW. The main feature characterizing A3a coccoliths was instead the nearly, or completely closed, central area; in this case, the ratio between SL and TW was very variable and not taken into account. Each coccolith was assigned to a single calcification variety and the relative abundance of each variety to the total *E*. *huxleyi* Type A coccoliths was calculated. The sum of A1, A2, A3a and A3b percentages in each sample is therefore equal to 100%. Our observations suggest that the norm is for a Type A coccosphere to be entirely formed of coccoliths of one calcification variety. However, a mix of calcification varieties was clearly identified on two coccosphere specimens from the SE Mediterranean (S3 Fig).

#### 2.2.3 Calcification index

The coccolith mass measured through SYRACO depends on both coccolith length and its degree of calcification. We obtained an indicator of the calcification degree, the calcification index C_i_, as an alternative to the “relative tube width” [61].

$$C_{i}=\frac{M_{s}}{M_{n}}$$ (1)

In Eq (1), M_s_ is the average coccolith mass measured with SYRACO for the sample under consideration, while M_n_ is the “normalized mass”. M_n_ was calculated based on [62], as in Eq (2):

$$M_{n}=V\times \rho_{c}$$ (2)

in which ρ_*c*_ is the density of calcite (= 2.7 pg μm^-3^) and V corresponds to the coccolith volume calculated as in Eq (3):

$$V=k_{s}\times L_{c}^{3}$$ (3)

where L_*c*_ corresponds to the average coccolith ‘corrected length’ obtained from SYRACO measurements (see next paragraph) and k_s_ is an estimated shape dependent constant (k_s_ = 0.02) for *E*. *huxleyi* type A normally calcified coccoliths [62].

We expected the coccolith distal shield length to be systematically underestimated by SYRACO. In fact, when the coccolith is observed in cross-polarized light, the calcitic coccolith tube appears extremely bright in comparison to the peripheral and thinner area of the shield [63]. To quantify this underestimation, we selected six samples from those already analyzed with SYRACO, so as to include a wide range of coccolith lengths. Fifty micrographs of flat-lying *E*. *huxleyi* Type A coccoliths were captured per sample, using the SEM at 30000 X magnification. Those images were analyzed with the open source software Fiji [64], a distribution of ImageJ [65]. The Coccobiom2 macro (http://ina.tmsoc.org/nannos/coccobiom/Usernotes.html; [61]), developed specifically for coccoliths, was chosen to facilitate the measurements. The image analysis done through this method allowed accurate measurements of the central area + tube length, and of the total distal shield length for each coccolith (S4 Fig). As expected, the coccolith lengths obtained from SYRACO were systematically greater than the SEM-derived sums of coccolith central area + tube length. Still, both of these quantities were smaller than the total coccolith lengths measured from SEM micrographs. The equation of the linear regression in the last plot of S4 Fig (y = 0.585 x + 0.4537) was used to correct the SYRACO-derived coccolith lengths of all remaining samples. As mentioned in the previous paragraph, the corrected length values were used in Eq (3).

### 2.3 Statistics

**Fig 3:**
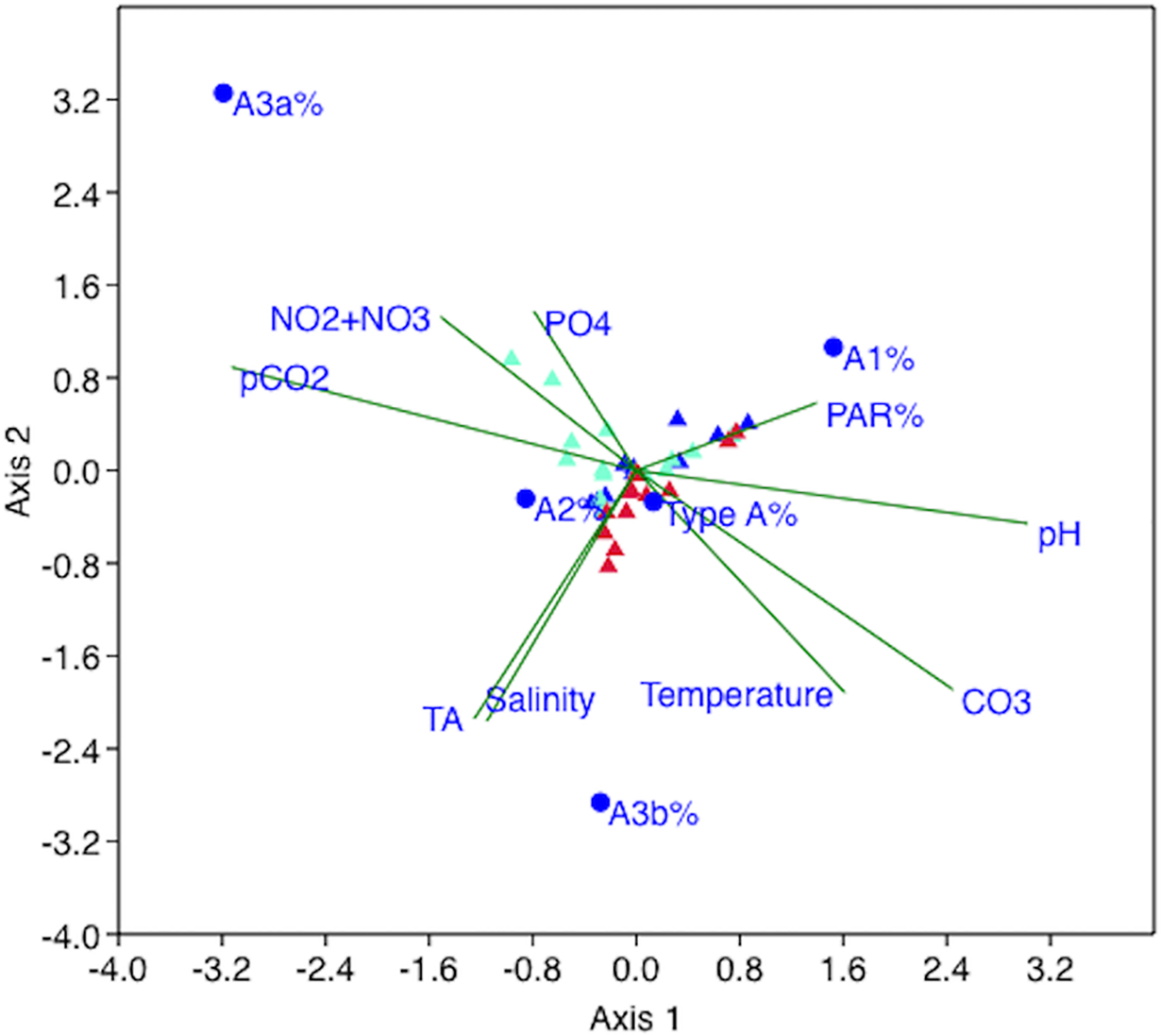
CCA biplot. Vectors radiating from the center symbolize the environmental gradients; their length is proportional to the strength of the gradient. The blue dots are the centroids of *E*. *huxleyi* Type A and of its four calcification varieties. The coloured triangles represent individual samples, differentiated by marine province (blue = A-G, aquamarine = SW Med., red = SE Med.).

A Canonical Correspondence Analysis (CCA) and a series of Spearman’s rank correlations were performed on the dataset. The CCA is a multivariate analysis [66], used here (Fig 3) to relate the observed *E*. *huxleyi* morphotypes and Type A calcification varieties distributions to the environmental gradients.

The Spearman’s analyses (Tables 2–4) tested the correlations between the morphological parameters (M_s_, L_c_, C_i_), the environmental variables, and the relative abundances of the *E*. *huxleyi* morphotypes and Type A calcification varieties (A1, A2, A3a, A3b); coefficients were regarded significant for *p* ≤ 0.05. The software PAST 3.14 [67] was used for these statistical tests. Regression analyses and correspondent coefficients (R^2^) were obtained plotting data in Grapher^TM^ 12 (Golden Software, LLC).

**Table 2:** Correlations among morphological parameters: Coccolith mass (M_s_), calcification index (C_i_) and corrected length (L_c_).

	M_s_	C_i_
C_i_	0.86**	
L_c_	0.91**	0.61**

Significant Spearman coefficients are in bold (N = 54; **p ≤ 0.01).

**Table 3:** Correlations between morphological parameters, Type A coccospheres % (with respect to the total *E*. *huxleyi* coccospheres; N = 52) and calcification varieties % (with respect to the total Type A coccoliths; N = 35).

	Type A%	A1%	A2%	A3a%	A3b%
M_s_	0.22	-0.33*	0.29	-0.03	0.75**
C_i_	0.03	-0.11	0.05	0.30	0.45**
L_c_	0.29*	-0.40*	0.39*	-0.28	0.81**

Significant Spearman coefficients are in bold (**p ≤ 0.01, *p ≤ 0.05).

**Table 4:** Environmental correlations of morphological parameters (N = 54), Type A coccospheres % (N = 52) and calcification varieties % (N = 35).

	M_s_	C_i_	L_c_	Type A%	A1%	A2%	A3a%	A3b%
Temperature	-0.28*	-0.47**	-0.11	0.32*	0.04	0.15	-0.73**	0.03
Salinity	0.61**	0.37**	0.65**	0.54**	-0.42**	0.19	-0.20	0.67**
Total alkalinity	0.57**	0.37**	0.60**	0.54**	-0.34*	0.10	-0.17	0.63**
pH	0.41**	0.29*	0.39**	0.53**	0.36*	-0.39*	-0.36*	0.16
*p*CO_2_	-0.42**	-0.25	-0.45**	-0.51**	-0.20	0.22	0.46**	-0.29
CO_3_^2-^	0.37**	0.13	0.47**	0.69**	0.10	-0.17	-0.55**	0.37*
NO_2_^-^+NO_3_^-^	-0.01	0.17	-0.08	-0.43**	0.16	-0.16	0.51**	-0.20
PO_4_^3-^	-0.12	0.10	-0.20	-0.46**	0.25	-0.15	0.44**	-0.33
PAR%	-0.54**	-0.44**	-0.47**	0.12	0.41	-0.21	-0.13	-0.57*

PAR% values were available only for the MedSeA cruise (N = 35 in relation to morphological parameters; N = 33 in relation to Type A coccospheres %; N = 16 in relation to calcification varieties %). Significant Spearman coefficients are in bold (**p ≤ 0.01, *p ≤ 0.05).

### 3 Results

Data from the Meteor M84/3 (April 2011) and MedSeA cruises (May 2013) have been combined to obtain the average values for all morphological variables, the relative abundances of the *E*. *huxleyi* morphotypes, and those of the Type A calcification varieties.

The CCA (Fig 3) revealed that 81.87% of the variance in the relative abundance of *E*. *huxleyi* Type A and of its four calcification varieties was explained by axis 1 (52.23%) and 2 (29.64%). Axis scores showed that, among the environmental parameters, the three major contributors to axis 1 were *p*CO_2_ (- 0.48), pH (0.47) and [CO_3_^2-^] (0.38); the three major contributors to axis 2 were instead salinity (- 0.33), total alkalinity (- 0.33) and temperature (- 0.30).

**Fig 4:**
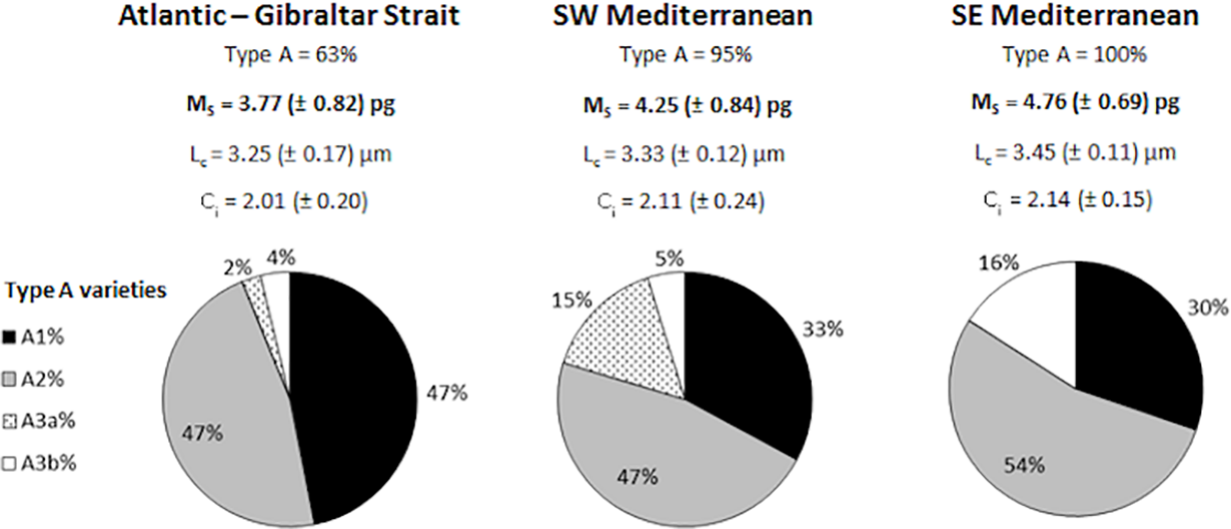
Longitudinal distribution of Type A morphotypes, average M_s_, L_c_, C_i_ and Type A calcification varieties. Data from the M84/3 and MedSeA cruises have been combined for each province presented in S2 Fig.

**Fig 5:**
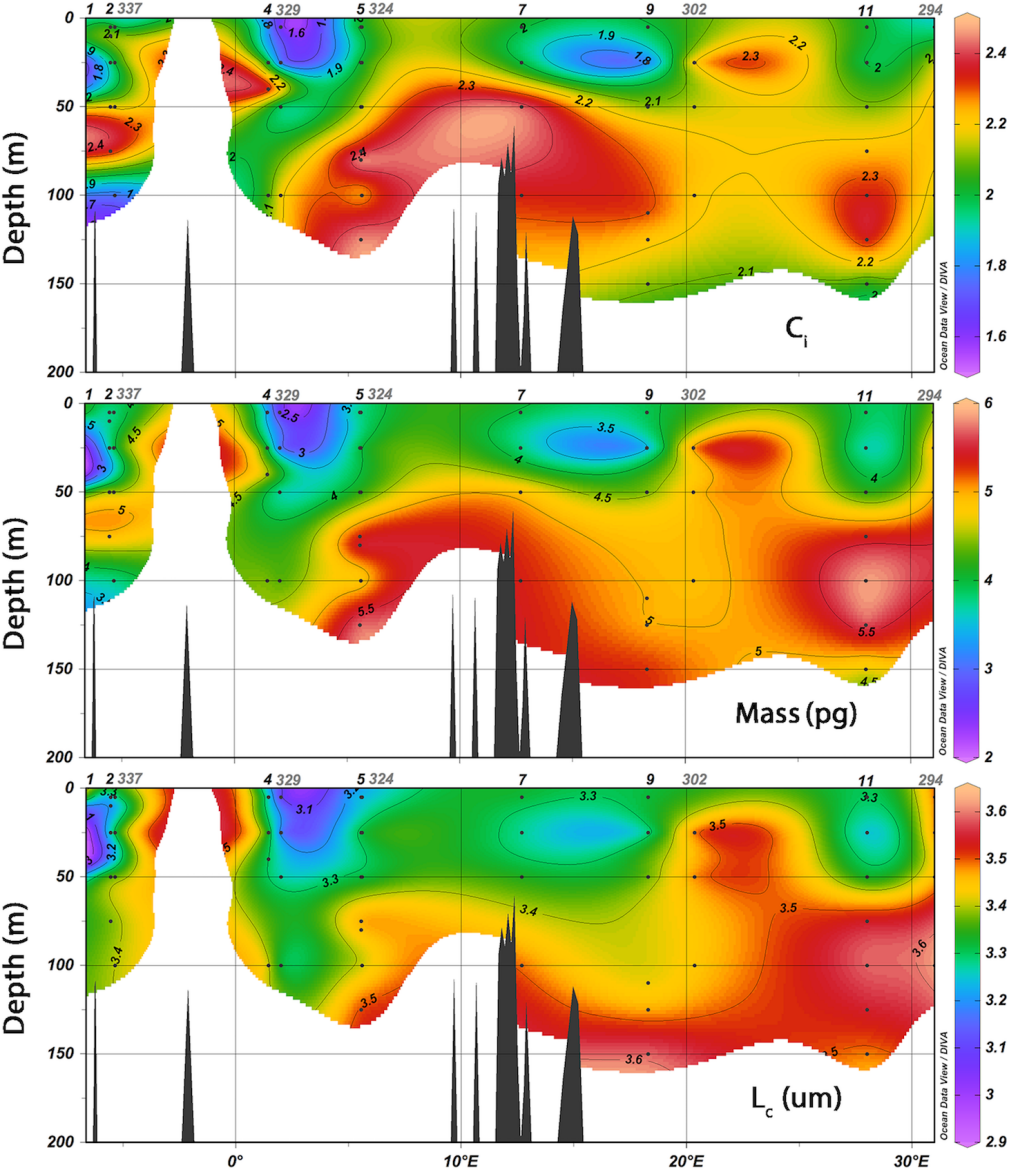
Coccolith calcification index (C_i_), mass (M_s_) and corrected length (L_c_) along the analyzed Mediterranean transect.

The average coccolith mass (M_s_) increased eastward between the Atlantic-Gibraltar Strait and the SE Mediterranean: from 3.77 to 4.76 pg. The average values of L_c_ and C_i_ increased in the same direction, respectively from 3.25 to 3.45 μm and from 2.01 to 2.14 (Fig 4). The lowest coccolith mass values were found in the upper photic zone of the Atlantic-Gibraltar Strait (minimum in station 1, at 40 m, 2.24 pg; Fig 5), while the highest values were found in the middle-lower photic zone of the SW and SE Mediterranean (maximum in station 11, at 100 m, 5.85 pg).

The largely dominant *E*. *huxleyi* morphotype in our samples was Type A (Fig 4). Type B/C, the only other morphotype of *E*. *huxleyi* observed, was poorly represented: on average, it corresponded to ≈ 5% of the total *E*. *huxleyi* coccospheres, except for St. 337 (the westernmost station sampled during the Meteor M84/3 cruise), where it reached ≈ 73%. Moreover, Type B/C coccospheres were almost never retrieved in the SE Mediterranean (0–1% of the total *E*. *huxleyi*).

A1 and A2 were the most abundant calcification varieties along the transect. The average percentage of A1 decreased from the Atlantic-Gibraltar Strait (47% of the total *E*. *huxleyi* Type A) towards the SE Mediterranean (30%), while A2 increased (from 47% to 54%). The high-calcified forms included A3a and A3b: A3a was present almost exclusively in the Atlantic-Gibraltar Strait province, at low relative abundance (2% of the *E*. *huxleyi* Type A coccoliths), and in the SW Mediterranean, where it reached up to 15%. Contrasting with the patchy distribution of A3a, A3b was found along the whole transect and increased in relative abundance eastwards, 4% to 16% (Fig 4).

**Fig 6:**
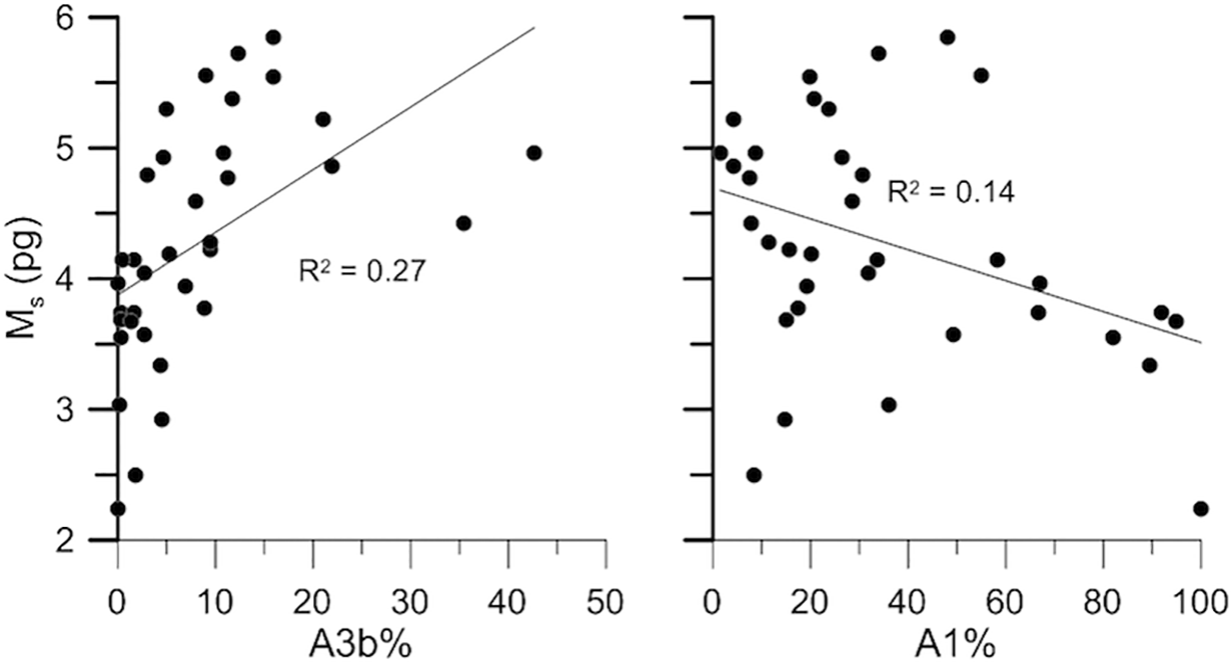
Relationships between coccolith mass (M_s_) and the percentages of A3b and A1. The percentages were calculated in respect to the absolute abundance of *E*. *huxleyi* Type A coccoliths. The black line represents the linear regression between the pairs of variables.

*Emiliania huxleyi* coccolith mass (M_s_) was positively correlated with C_i_ and L_c_ (S5 Fig, Table 2), and with the relative abundance of A3b (Fig 6, Table 3); while it was negatively correlated with the relative abundance of A1 (Fig 6, Table 3). Coccolith length (L_c_) had similar coefficients with respect to A1 and A3b, but was also significantly correlated with A2; C_i_ was correlated only with A3b. Other significant correlations were found between morphological and environmental parameters (Table 4; S6 Fig): M_s_, C_i_ and L_c_ were all strongly correlated with the salinity, total alkalinity, pH and Photosynthetically Active Radiation (PAR%). Weaker but statistically significant relationships with *p*CO_2_, [CO_3_^2-^], temperature and dissolved oxygen were also found. The relative abundance of Type A coccospheres showed the strongest correlations with the carbonate system, salinity and nutrients, followed by temperature (Table 4; S7 Fig). All of the Type A calcification varieties displayed significant correlations with at least one carbonate system parameter. Furthermore, the calcification variety A1 was significantly correlated with salinity, A3a with temperature and nutrient concentrations, A3b with salinity and PAR% (Table 4).

## 4 Discussion

### 4.1 Morphological controls over *E*. *huxleyi* coccolith mass

Along the studied transect, the average *E*. *huxleyi* coccolith mass per sample oscillated between 2.24 and 5.85 pg. This range of values is similar to that registered in sediment trap material from the NW Mediterranean (2.81 − 5.61 pg; [68]) and is overall higher than that measured in surface sediments from the South Atlantic-southwestern Indian Ocean (1.73–4.85 pg; [21]). The largest variations in average *E*. *huxleyi* coccolith mass should mainly reflect the geographical distribution of its morphotypes [12]. Still, in oceanic regions where one morphotype is largely predominant, as in the Mediterranean Sea [46,47,49], the calcification varieties of that particular morphotype can be expected to exercise a major control.

We verified that Type A was by far the dominant morphotype along the studied Mediterranean transect (Fig 4), as in [49,60,69]. The concomitance of high Type B/C percentages and of low average coccolith mass values in the Atlantic-Gibraltar province is likely not a coincidence: Type B/C is typically less calcified than Type A. Still, the Spearman analysis did not reveal any significant correlation between coccolith mass and the percentage of Type A coccospheres with respect to the total *E*. *huxleyi* population (Table 3). Our results suggest instead that most of coccolith mass variability in the Mediterranean Sea is actually controlled by the relative abundance of the Type A calcification varieties. The significance of the correlations between A1, A3b and the average coccolith mass, indicates that our morphological subdivision of Type A into calcification varieties is of relevance to addressing coccolith calcite content. Moreover, we can infer that the high-calcified A3b coccoliths were usually larger than those of other calcification varieties, especially if compared to the low-calcified A1 coccoliths. Similar positive trends between size and calcification degree were noticed in oceanic communities of the North Atlantic [61]. Overall, the comparison between morphological variables suggests that *E*. *huxleyi* coccolith mass is controlled by the distribution of the Type A calcification varieties, characterized by different size ranges and calcification degrees. The Eastern Mediterranean was richer in large, high-calcified specimens (A3b), explaining the high coccolith mass values obtained from this province (Figs 4 and 5). Coccoliths of A3a were present almost exclusively in the Western Mediterranean, where they occasionally became more abundant than A3b (in stations 2, 4, 7, 324 and 329). The two high-calcified varieties A3a and A3b should have both contributed positively to the average calcification degree. However, increases in the relative abundance of A3a did not always correspond to increases in the average coccolith length. For example, in station 329, A3a increased from 22 to 58%, while the average *E*. *huxleyi* coccolith length decreased from 3.30 to 3.27 μm: A3a might actually contain less calcite than A3b, due to its smaller average size, resulting in a lower mass. We conclude that the contribution of A3a to the average coccolith calcification degree, length, and ultimately mass, was negligible at the time of sampling, both at basin (whole W-E transect) and province (SW Mediterranean) scale. The calcification varieties A1 and A3b, instead, controlled most of the *E*. *huxleyi* coccolith mass variability along the transect.

### 4.2 Ecology of *E*. *huxleyi* morphotypes and Type A calcification varieties

*Emiliania huxleyi* morphotypes are genetically-controlled and have distinct environmental preferences [14,49,70,71]. For example, Type B/C commonly lives in cold waters, rich in nutrients and with low calcite saturation state. These preferences explain the limited distribution of Type B/C in the Mediterranean Sea compared to Type A, especially in the Eastern province (Fig 4); the high percentages of Type B/C in the Atlantic−Gibraltar province have also been interpreted as a biological signal of the Atlantic water inflow into the Mediterranean Sea [49].

The distribution of the Type A calcification varieties seems to be controlled by an array of environmental parameters: carbonate chemistry, salinity, temperature and nutrient concentrations (Fig 3, Table 4). However, It is not clear if this distribution reflects their ecological affinities or their phenotypic plasticity. With the currently available data, it is difficult to determine if the Type A calcification varieties are characterized by distinct genotypes or not.

Previous field observations have demonstrated the existence of a connection between multiple environmental parameters and the calcification degree of *E*. *huxleyi* populations [12,14,38,46,47,58]. Besides, distinct sets of environmental parameters appear to control the average coccolith mass, depending on the oceanic region [21].

Our results suggest that carbonate chemistry could have an important role in regulating the distribution of the Type A calcification varieties (Table 4, S7 Fig). However, there is not a straightforward relationship between the calcification degree of Type A coccoliths and the carbonate parameters: the distributions of the two high-calcified varieties (A3a and A3b) were related differently to total alkalinity, pH, *p*CO_2_ and especially in respect to [CO_3_^2-^]. This is in accord with previous field studies [12,38], showing that low-calcified and high-calcified specimens can both occur in waters having low pH, low calcite saturation state and low [CO_3_^2-^]. Hence, the significant correlation we observed between the calcification degree (C_i_) and water pH (Table 4; S6 Fig) should be evaluated with caution.

The distributions of the A1 and A3b varieties followed, in addition to carbonate chemistry, the salinity gradients; in the case of A3b, we also observed an inverse relationship with PAR% (Fig 3, Table 4). Overall, A1 was predominantly encountered in the upper photic zone of the Atlantic-Gibraltar Strait and SW Mediterranean, characterized by low salinity waters; while A3b was mainly found in the middle-lower photic zone of the SE Mediterranean, characterized by high salinity waters. A similar vertical distribution of the calcification varieties was observed, at smaller scale, in the NE Aegean Sea [47]: low-calcified *E*. *huxleyi* Type A coccoliths were particularly abundant in the upper 20 m of the water column, within the low salinity-low density layer of inflowing Black Sea water. The calcification varieties’ distribution observed in our samples could thus depend on the salinity preferences of the strains and/or could be regulated by physical factors (density and vertical stratification of water masses).

Temperature and nutrient concentration seem to exercise a major control on the distribution of A3a coccoliths (Fig 3, Table 4). Our correlations can help explain a relative increase in high-calcified coccoliths during winter-spring in the Aegean Sea, when temperatures were lower and nutrient concentrations higher [46,47].

As anticipated, we provide two interpretations for the existence of the *E*. *huxleyi* Type A calcification varieties: each calcification variety could represent a population characterized by (a) one genotype, or (b) multiple genotypes.

The first interpretation contemplates the potential production of several calcification varieties during the lifespan of a single *E*. *huxleyi* cell, triggered by changing environmental conditions. In support of this hypothesis, we report the occurrence of seasonal unimodal patterns of calcification in the Aegean Sea [46] and of occasional ‘mixed’ coccospheres, composed of multiple calcification varieties, retrieved along our Mediterranean transect (S3 Fig) and in the Atlantic Ocean [38]. Furthermore, laboratory experiments have demonstrated that the calcification process in *E*. *huxleyi* can be strongly influenced by carbonate chemistry [8], salinity [24,72–74], temperature [22,75] and nutrient concentrations [23]: the environmental conditions regulate the cellular energetic consumption dedicated to the calcification process. A complication is that, in laboratory and field studies, two different concepts of ‘calcification’ are used: calcification rate and calcification degree. During cultures, PIC production and PIC: POC ratio are obtained, while in filtered marine water samples and sediments it is necessary to refer to coccolith calcite mass, degree of calcification and/or size. Recent work [22] however, indicates that high coccolith mass for *E*. *huxleyi* might be a good indicator of high PIC production.

The second interpretation envisages the idea that each calcification variety represents a group of strains adapted to a specific ecological niche. In this case, the correlations observed in our samples (Table 4) would indicate the ecological preferences of each calcification variety. *Emiliania huxleyi* has a high genetic diversity in today’s oceans, as proved by both microsatellite and laboratory data [12,18,76]. Although the relationship between *E*. *huxleyi* genome and its morphotypes is not straightforward [18], it has been observed that shifts in the relative abundance of morphotypes generally correspond to shifts in the relative abundance of distinct genetic codes [12]. Such genetic diversity translates into strain-specific responses to environmental change [23,77]: at least two clades of *E*. *huxleyi* Type A, characterized by different preferential habitats, are present in the Mediterranean Sea; temperature and phosphate are likely the most important environmental constrains on their distribution [17,18]. Another interesting point raised by a recent study is that inter-strain genetic variability can potentially induce larger phenotypic differences than phenotypic plasticity itself [78].

Although the occurrence of ‘mixed’ coccospheres would induce a quick conclusion (i.e. each Type A cell can produce all four calcification varieties), we cannot exclude the possibility that those rare specimens are artefacts: a xenosphere formed by a mix *E*. *huxleyi* coccolith morphologies, similarly to the specimens observed in our samples and in the Atlantic Ocean [38], was identified by [79] as a probable artefact.

The four calcification varieties described in this work should be ideally isolated and subjected to controlled environmental perturbations. That would be a direct way to assess their nature and their specific responses to environmental change. Among the four calcification varieties, A3a has a characteristic distribution in the SW Mediterranean and a peculiar affinity for low temperatures-high nutrients, making it a very interesting candidate to test in the future.

Under climate change, we can expect shifts within the *E*. *huxleyi* populations. The Mediterranean Sea could experience not only a shift in the proportion of *E*. *huxleyi* morphotypes (Type A vs. Type B/C), but also in the proportion of the Type A calcification varieties (A1, A2, A3a, A3b). The average *E*. *huxleyi* coccolith mass will depend mainly on the response of the Type A calcification varieties. Based on their present distributions, we foresee that A3a will likely become even rarer under warming and enhanced water column stratification. The fate of A1 and A3b could be instead mainly linked to carbonate chemistry and salinity, but the relative importance of these parameters cannot be discerned with certainty based only on available data. Overall, the proportion of A1 and A3b calcification varieties is expected to retain the greatest control over the average *E*. *huxleyi* coccolith mass in the Mediterranean Sea.

## 5 Conclusions

*Emiliania huxleyi* coccolith mass variability in the Mediterranean Sea is primarily modulated by the relative abundance of Type A calcification varieties, being Type A the largely dominant *E*. *huxleyi* morphotype in this oceanographic region. The large, high-calcified specimens (A3b) are more common in the SE basin, while the small, low-calcified specimens (A1) are more common in the Atlantic-Gibralatar Strait and in the SW basin. Our results indicate that seawater carbonate chemistry, water salinity, temperature and nutrient concentrations might all contribute in regulating the distribution of different *E*. *huxleyi* Type A calcification varieties. The nature of the calcification varieties observed is possibly linked to the cellular energetic consumption in relation to the calcification process in different environmental settings. Overall, they might represent a mix of genotypes. The available data suggest that *E*. *huxleyi* Type B/C and the Type A calcification variety A3a will become rarer in the Mediterranean Sea under warming and, especially, under enhanced water column stratification. The average *E*. *huxleyi* coccolith mass in the Mediterranean Sea is basically controlled by the proportion of the Type A calcification varieties A1 and A3b. The fate of A1 and A3b is probably closely linked to changes in seawater carbonate chemistry and needs to be assessed through laboratory perturbation experiments.

## Supporting information

S1 Fig Total alkalinity along the studied transect, during the Meteor M84/3 cruise and the MedSeA cruise. (TIF)

S2 Fig Main water masses within the upper 200 m during the MedSeA and M84/3 cruises. A-G = Atlantic Water from the eastern Atlantic Ocean and the Gibraltar Strait; SW Med. = Modified Atlantic Water from the South Western Mediterranean; SE Med. = Modified Atlantic Water from the South Eastern Mediterranean.
(TIF)

S3 Fig *Emiliania huxleyi* Type A coccospheres composed by a mix of coccolith calcification varieties. Blue = A1, green = A3b, orange = A3a. Specimens are from St. 11, collected at 75 m and 100 m depth.
(TIF)

S4 Fig Coccolith morphometry and correspondent standard deviations for six samples: Comparisons between SYRACO results and those obtained from the analysis of SEM micrographs. The linear regression function in the last plot was used to calculate the average corrected length (L_c_) for all samples.
(TIF)

S5 Fig Linear regressions between coccolith mass (M_s_), corrected length (L_c_) and calcification index (C_i_). (TIF)

S6 Fig Plots combining the morphological and carbonate system parameters. Linear regressions are shown only for combinations of parameters which have significant Spearman correlation coefficients (p ≤ 0.05).
(TIF)

S7 Fig Plots combining the percentage of low-calcified / high-calcified Type A varieties and the carbonate system parameters. Linear regressions are shown only for combinations of parameteres which have significant Spearman correlation coefficients (p ≤ 0.05).
(TIF)

S1 Table Comparison between the *E*. *huxleyi* Type A calcification varieties defined in this work (A1, A3a, A3b) and those appeared in the literature concerning the North Atlantic Ocean and the Mediterranean Sea (Med). The variety A2 is not included being very common (showing no special patterns in our samples) and medium-calcified.
(DOCX)

## References

[1] IPCC. Climate Change 2013: The Physical Science Basis. Contribution of Working Group I to the Fifth Assessment Report of the Intergovernmental Panel on Climate Change Cambridge, United Kingdom and New York, NY, USA: Cambridge University Press; 2013.

[2] Orr JC, Fabry VJ, Aumont O, Bopp L, Doney SC, Feely RA, et al Anthropogenic ocean acidification over the twenty-first century and its impact on calcifying organisms. Nature. 2005;437:681–686. 10.1038/nature04095 16193043

[3] Kroeker KJ, Kordas RL, Crim R, Hendriks IE, Ramajo L, Singh GS, et al Impacts of ocean acidification on marine organisms: Quantifying sensitivities and interaction with warming. Glob Chang Biol. 2013;19:1884–1896. 10.1111/gcb.12179 23505245 PMC3664023

[4] Portner H-O, Karl DM, Boyd PW, Cheung WWL, Lluch-Cota SE, Nojiri Y, et al Ocean systems In: Field CB, Barros VR, Dokken DJ, Mach KJ, Mastrandrea MD, Bilir TE, et al, editors. Climate Change 2014: Impacts, Adaptation, and Vulnerability. Part A: Global and Sectoral Aspects. Contribution of Working Group II to the Fifth Assessment Report of the Intergovernmental Panel on Climate Change Cambridge, United Kingdom and New York, NY, USA: Cambridge University Press; 2014 pp. 411–484.

[5] Honjo S, Manganin SJ, Cole JJ. Sedimentation of biogenic matter in the deep ocean. Deep Sea Res Part A, Oceanogr Res Pap. 1982;29:609–625.

[6] Winter A, Siesser WG. Coccolithophores New York: Cambridge University Press; 1994.

[7] Broecker W, Clark E. Ratio of coccolith CaCO_3_ to foraminifera CaCO_3_ in late Holocene deep sea sediments. Paleoceanography. 2009;24:1–11.

[8] Meyer J, Riebesell U. Reviews and Synthesis: Responses of coccolithophores to ocean acidification: a meta-analysis. Biogeosciences. 2015;12:1671–1682.

[9] Jin P, Gao K. Reduced resilience of a globally distributed coccolithophore to ocean acidification: Confirmed up to 2000 generations. Mar Pollut Bull. 2016;103:101–108. 10.1016/j.marpolbul.2015.12.039 26746379

[10] Müller MN, Trull TW, Hallegraeff GM. Independence of nutrient limitation and carbon dioxide impacts on the Southern Ocean coccolithophore *Emiliania huxleyi*. The ISME Journal. 2017;11:1777–1787. 10.1038/ismej.2017.53 28430186 PMC5520040

[11] Milner S, Langer G, Grelaud M, Ziveri P. Ocean warming modulates the effects of acidification on *Emiliania huxleyi* calcification and sinking. Limnol Oceanogr. 2016;61:1322–1336.

[12] Beaufort L, Probert I, de Garidel-Thoron T, Bendif EM, Ruiz-Pino D, Metzl N, et al Sensitivity of coccolithophores to carbonate chemistry and ocean acidification. Nature. 2011;476:80–83. 10.1038/nature10295 21814280

[13] Rickaby REM, Hermoso M, Lee RBY, Rae BD, Heureux AMC, Balestreri C, et al Environmental carbonate chemistry selects for phenotype of recently isolated strains of *Emiliania huxleyi*. Deep Res Part II. 2016;127:28–40.

[14] Cubillos JC, Wright SW, Nash G, de Salas MF, Griffiths B, Tilbrook B, et al Calcification morphotypes of the coccolithophorid *Emiliania huxleyi* in the Southern Ocean: changes in 2001 to 2006 compared to historical data. Mar Ecol Prog Ser. 2007;348:47–54.

[15] Hoppe CJM, Langer G, Rost B. *Emiliania huxleyi* shows identical responses to elevated *p*CO2 in TA and DIC manipulations. J Exp Mar Bio Ecol. 2011;406:54–62.

[16] Langer G, Probert I, Nehrke G, Ziveri P. The morphological response of *Emiliania huxleyi* to seawater carbonate chemistry changes: an inter-strain comparison. J Nannoplankt Res. 2011;32:29–34.

[17] Bendif EM, Probert I, Carmichael M, Romac S, Hagino K, de Vargas C. Genetic delineation between and within the widespread coccolithophore morpho-species *Emiliania huxleyi* and *Gephyrocapsa oceanica* (Haptophyta). J Phycol. 2014;50:140–148. 10.1111/jpy.12147 26988015

[18] Hagino K, Bendif EM, Young JR, Kogame K, Probert I, Takano Y, et al New evidence for morphological and genetic variation in the cosmopolitan coccolithophore *Emiliania huxleyi* (Prymnesiophyceae) from the COX1b-ATP4 genes. J Phycol. 2011;47:1164–1176. 10.1111/j.1529-8817.2011.01053.x 27020197

[19] Read BA, Kegel J, Klute MJ, Kuo A, Lefebvre SC, Maumus F, et al Pan genome of the phytoplankton *Emiliania* underpins its global distribution. Nature. 2013;499:209–213. 10.1038/nature12221 23760476

[20] von Dassow P, John U, Ogata H, Probert I, Bendif EM, Kegel JU, et al Life-cycle modification in open oceans accounts for genome variability in a cosmopolitan phytoplankton. The ISME Journal. 2015;9:1365–1377. 10.1038/ismej.2014.221 25461969 PMC4438323

[21] Horigome MT, Ziveri P, Grelaud M, Baumann K-H, Marino G, Mortyn PG. Environmental controls on the *Emiliania huxleyi* calcite mass. Biogeosciences. 2014;11:2295–2308.

[22] Rosas-Navarro A, Langer G, Ziveri P. Temperature affects the morphology and calcification of *Emiliania huxleyi* strains. Biogeosciences. 2016;13:2913–2926.

[23] Oviedo AM, Langer G, Ziveri P. Effect of phosphorus limitation on coccolith morphology and element ratios in Mediterranean strains of the coccolithophore *Emiliania huxleyi*. J Exp Mar Bio Ecol. 2014;459:105–113.

[24] Saruwatari K, Satoh M, Harada N, Suzuki I, Shiraiwa Y. Change in coccolith size and morphology due to response to temperature and salinity in coccolithophore *Emiliania huxleyi* (Haptophyta) isolated from the Bering and Chukchi seas. Biogeosciences. 2016;13:2743–2755.

[25] Young JR, Westbroek P. Genotypic variation in the coccolithophorid species *Emiliania huxleyi*. Mar Micropaleontol. 1991;18:5–23.

[26] van Bleijswijk J, van der Wal P, Kempers R, Veldhuis M, Young JR, Muyzer G, et al Distribution of two types *Emiliania huxleyi* (Prymnesiophyceae) in the norteastern Atlantic region as determined by immunofluorescence and coccolith morphology. J Phycol. 1991;27:566–570.

[27] Cook SS, Whittock L, Wright SW, Hallegraeff GM. Photosynthetic pigment and genetic differences between two Southern Ocean morphotypes of *Emiliania huxleyi* (Haptophyta). J Phycol. 2011;47:615–626. 10.1111/j.1529-8817.2011.00992.x 27021991

[28] Hagino K, Okada H, Matsuoka H. Coccolithophore assemblages and morphotypes of *Emiliania huxleyi* in the boundary zone between the cold Oyashio and warm Kuroshio currents off the coast of Japan. Mar Micropaleontol. 2005;55:19–47.

[29] Medlin LK, Barker GLA, Campbell L, Green JC, Hayes PK, Marie D, et al Genetic characterisation of *Emiliania huxleyi* (Haptophyta). J Mar Syst. 1996;9:13–31.

[30] Paasche E. A review of the coccolithophorid *Emiliania huxleyi* (Prymnesiophyceae), with particular reference to growth, coccolith formation, and calcification-photosynthesis interactions. Phycologia. 2002;40:503–529.

[31] Schroeder D, Biggi GF, Hall M, Davy J, Martínez JM, Richardson AJ, et al A genetic marker to separate *Emiliania huxleyi* (Prymnesiophyceae) morphotypes. J Phycol. 2005;41:874–879.

[32] McIntyre A, Bé AWH. Modern coccolithophoridae of the Atlantic Ocean—I. Placoliths and cyrtoliths. Deep Sea Res Oceanogr Abstr. 1967;14(5):561–597.

[33] Poulton AJ, Young JR, Bates NR, Balch WM. Biometry of detached *Emiliania huxleyi* coccoliths along the Patagonian Shelf. Mar Ecol Prog Ser. 2011;443:1–17.

[34] Ziveri P, Thunell RC. Coccolithophore export production in Guaymas Basin, Gulf of California: response to climate forcing. Deep Res Part II. 2000;47:2073–2100.

[35] Young JR, Geisen M, Cros L, Kleijne A, Sprengel C, Probert I, et al A guide to extant coccolithophore taxonomy Journal of Nannoplankton Research Special Issue. Bremerhaven: International Nannoplankton Association; 2003.

[36] Young JR, Bown PR, Lees JA. Nannotax3 website International Nannoplankton Association Accessed on 2017 Available from: http://www.mikrotax.org/Nannotax3.

[37] Beaufort L, Heussner S. Seasonal dynamics of calcareous nannoplankton on a West European continental margin: the Bay of Biscay. Mar Micropaleontol. 2001;43:27–55.

[38] Smith HEK, Tyrrell T, Charalampopoulou A, Dumousseaud C, Legge OJ, Birchenough S, et al Predominance of heavily calcified coccolithophores at low CaCO_3_ saturation during winter in the Bay of Biscay. PNAS. 2012;109:8845–8849. 10.1073/pnas.1117508109 22615387 PMC3384182

[39] Berger C, Meier KJS, Kinkel H, Baumann K-H. Changes in calcification of coccoliths under stable atmospheric CO_2_. Biogeosciences. 2014;11:929–944.

[40] Ziveri P, Thunell RC, Rio D. Export production of coccolithophores in an upwelling region: Results from San Pedro Basin, Southern California Borderlands. Mar Micropaleontol. 1995;24:335–358.

[41] Alcober J, Castelló M, Gomis C. Polimorfismo de *Emiliania huxleyi* (Loh.) Hay & Mohler en las aguas de los alrededores de la Isla de Tabarca (Alicante). Stud Bot. 1994;13:61–64.

[42] Cros L, Fortuño J-M. Atlas of northwestern Mediterranean coccolithophores. Sci 3 2002;66:1–186.

[43] Dimiza MD, Triantaphyllou M V., Krasakopoulou E. Coccolithophores (calcareous nannoplankton) distribution in the surface waters of the western Cretan Straits (South Aegean Sea): productivity and relation with the circulation pattern. Hell J Geosci. 2008;45:55–64.

[44] Dimiza MD, Triantaphyllou M V., Dermitzakis MD. Seasonality and ecology of living coccolithophores in Eastern Mediterranean coastal environments (Andros Island, Middle Aegean Sea). Micropaleontology. 2008;54:159–175.

[45] Riaux-Gobin C, Chrétiennot-Dinet M-J, Descolas-Gros C. Undamaged sedimented coccolithophorids in a deep environment (continental slope of the Gulf of Lions). Mar Geol. 1995;123:239–252.

[46] Triantaphyllou M V., Dimiza MD, Krasakopoulou E, Malinverno E, Lianou V, Souvermezoglou E. Seasonal variation in *Emiliania huxleyi* coccolith morphology and calcification in the Aegean Sea (Eastern Mediterranean). Geobios. 2010;43:99–110.

[47] Karatsolis BT, Triantaphyllou M V., Dimiza MD, Malinverno E, Lagaria A, Mara P, et al Coccolithophore assemblage response to Black Sea Water inflow into the North Aegean Sea (NE Mediterranean). Cont Shelf Res. 2016;149:138–150.

[48] Kershaw S. Oceanography: an Earth Science Perspective Cheltneham: Stanley Thornes Ltd; 2013.

[49] Oviedo A, Ziveri P, Álvarez M, Tanhua T. Is coccolithophore distribution in the Mediterranean Sea related to seawater carbonate chemistry? Ocean Sci. 2015;11:13–32.

[50] D’Amario B, Ziveri P, Grelaud M, Oviedo A, Kralj M. Coccolithophore haploid and diploid distribution patterns in the Mediterranean Sea: Can a haplo-diploid life cycle be advantageous under climate change? J Plankton Res. 2017;39:781–794.

[51] Hainbucher D, Rubino A, Cardin V, Tanhua T, Schroeder K, Bensi M. Hydrographic situation during cruise M84/3 and P414 (spring 2011) in the Mediterranean Sea. Ocean Sci Discuss. 2013;10:2399–2432.

[52] Tanhua T, Hainbucher D, Cardin V, Álvarez M, Civitarese G, McNichol a. P, et al Repeat hydrography in the Mediterranean Sea, data from the *Meteor* cruise 84/3 in 2011. Earth Syst Sci Data. 2013;5:289–294.

[53] Dollfus D, Beaufort L. Fat neural network for recognition of position-normalised objects. Neural Networks. 1999;12:553–560. 12662696 10.1016/s0893-6080(99)00011-8

[54] Beaufort L, Dollfus D. Automatic recognition of coccoliths by dynamical neural networks. Mar Micropaleontol. 2004;51:57–73.

[55] Beaufort L. Weight estimates of coccoliths using the optical properties (birefringence) of calcite. Micropaleontology. 2005;51:289–298.

[56] Incarbona A, Di Stefano E, Patti B, Pelosi N, Bonomo S, Mazzola S, et al Holocene millennial-scale productivity variations in the Sicily Channel (Mediterranean Sea). Paleoceanography. 2008;23:1–18.

[57] Incarbona A, Ziveri P, Di Stefano E, Lirer F, Mortyn G, Patti B, et al The impact of the little ice age on coccolithophores in the central mediterranea sea. Clim Past. 2010;6:795–805.

[58] Flores J-A, Sierro FJ, Francés G, Vazquez A, Zamarreno I. The last 100,000 years in the western Mediterranean: sea surface water and frontal dynamics as revealed by coccolithophores. Mar Micropaleontol. 1997;29:351–66.

[59] Bonomo S, Cascella A, Alberico I, Sorgato S, Pelosi N, Ferraro L, et al Reworked Coccoliths as runoff proxy for the last 400 years: The case of Gaeta Gulf (central Tyrrhenian Sea, Central Italy). Palaeogeogr Palaeoclimatol Palaeoecol. 2016;459:15–28.

[60] Knappertsbusch M. Geographic distribution of living and Holocene coccolithophores in the Mediterranean Sea. Mar Micropaleontol. 1993;21:219–247.

[61] Young JR, Poulton AJ, Tyrrell T. Morphology of *Emiliania huxleyi* coccoliths on the northwestern European shelf—is there an influence of carbonate chemistry? Biogeosciences. 2014;11:4771–4782.

[62] Young JR, Ziveri P. Calculation of coccolith volume and it use in calibration of carbonate flux estimates. Deep Sea Res Part II Top Stud Oceanogr. 2000;47:1679–1700.

[63] Beaufort L, Couapel M, Buchet N, Claustre H, Goyet C. Calcite production by coccolithophores in the south east Pacific Ocean. Biogeosciences. 2008;5:1101–1117.

[64] Schindelin J, Arganda-Carreras I, Frise E, Kaynig V, Longair M, Pietzsch T, et al Fiji: an open-source platform for biological-image analysis. Nat Methods. 2012;9:676–682. 10.1038/nmeth.2019 22743772 PMC3855844

[65] Schneider C A, Rasband WS, Eliceiri KW. NIH Image to ImageJ: 25 years of image analysis. Nat Methods. Nature Publishing Group; 2012;9:671–675. 22930834 10.1038/nmeth.2089 PMC5554542

[66] Palmer MW. Putting things in even better order: the advantages of canonical correspondence analysis. Ecology. 1993;74:2215–2230.

[67] Hammer Ø, Harper DAT, Ryan PD. Paleontological statistics software package for education and data analysis. Palaeontol Electron. 2001;4:9–18.

[68] Meier KJS, Beaufort L, Heussner S, Ziveri P. The role of ocean acidification in *Emiliania huxleyi* coccolith thinning in the Mediterranean Sea. Biogeosciences. 2014;11:2857–2869.

[69] Ziveri P, Broerse ATC, van Hinte JE, Westbroek P, Honjo S. The fate of coccoliths at 48°N 21°W, Northeastern Atlantic. Deep Sea Res Part II Top Stud Oceanogr. 2000;47:1853–1875.

[70] Henderiks J, Winter A, Elbrächter M, Feistel R, van der Plas A, Nausch G, et al Environmental controls on *Emiliania huxleyi* morphotypes in the Benguela coastal upwelling system (SE Atlantic). Mar Ecol Prog Ser. 2012;448:51–66.

[71] Hagino K, Okada H, Matsuoka H. Coccolithophore assemblages and morphotypes of *Emiliania huxleyi* in the boundary zone between the cold Oyashio and warm Kuroshio currents off the coast of Japan. Mar Micropaleontol. 2005;55:19–47.

[72] Fielding SR, Herrle JO, Bollmann J, Worden RH, Montagnes DJS. Assessing the applicability of *Emiliania huxleyi* coccolith morphology as a sea-surface salinity proxy. Limnol Oceanogr. 2009;54:1475–80.

[73] Green JC, Heimdal BR, Paasche E, Moate R. Changes in calcification and the dimensions of coccoliths of *Emiliania huxleyi* (Haptophyta) grown at reduced salinities. Phycologia. 1998;37:121–131.

[74] Paasche E, Brubak S, Skattebøl S, Young JR, Green JC. Growth and calcification in the coccolithophorid *Emiliania huxleyi* (Haptophyceae) at low salinities. Phycologia. 1996;35:394–403.

[75] Sett S, Bach LT, Schulz KG, Koch-Klavsen S, Lebrato M, Riebesell U. Temperature modulates coccolithophorid sensitivity of growth, photosynthesis and calcification to increasing seawater *p*CO2. PLoS ONE. 2014;9:e88308 10.1371/journal.pone.0088308 24505472 PMC3914986

[76] Iglesias-Rodríguez MD, Schofield OM, Batley J, Medlin LK, Hayes PK. Intraspecific genetic diversity in the marine coccolithophore *Emiliania huxleyi* (Prymnesiophyceae): the use of microsatellite analysis in marine phytoplankton population studies. J Phycol. 2006;42:526–536.

[77] Zhang Y, Bach LT, Lohbeck KT, Schulz KG, Listmann L, Klapper R, et al Population-specific responses in physiological rates of *Emiliania huxleyi* to a broad CO_2_ range. Biogeosciences Discuss. 2018; 10.5194/bg-2018-47

[78] Blanco-Ameijeiras S, Lebrato M, Stoll HM, Iglesias-Rodriguez D, Müller MN, Méndez-Vicente A, et al Phenotypic variability in the coccolithophore *Emiliania huxleyi*. PLoS ONE. 2016;11:e0157697 10.1371/journal.pone.0157697 27348427 PMC4922559

[79] Young JR, Geisen M. Xenospheres—Associations of coccoliths resembling coccospheres. J Nannoplankt Res. 2002;24:27–35.

