# Supplementary figures and images for: Emiliania huxleyi coccolith calcite mass modulation by morphological changes and ecology in the Mediterranean Sea
Source: PLoS One. 2018 Jul 24;13(7):e0201161. doi: 10.1371/journal.pone.0201161 (PMC6057672; doi:10.1371/journal.pone.0201161)

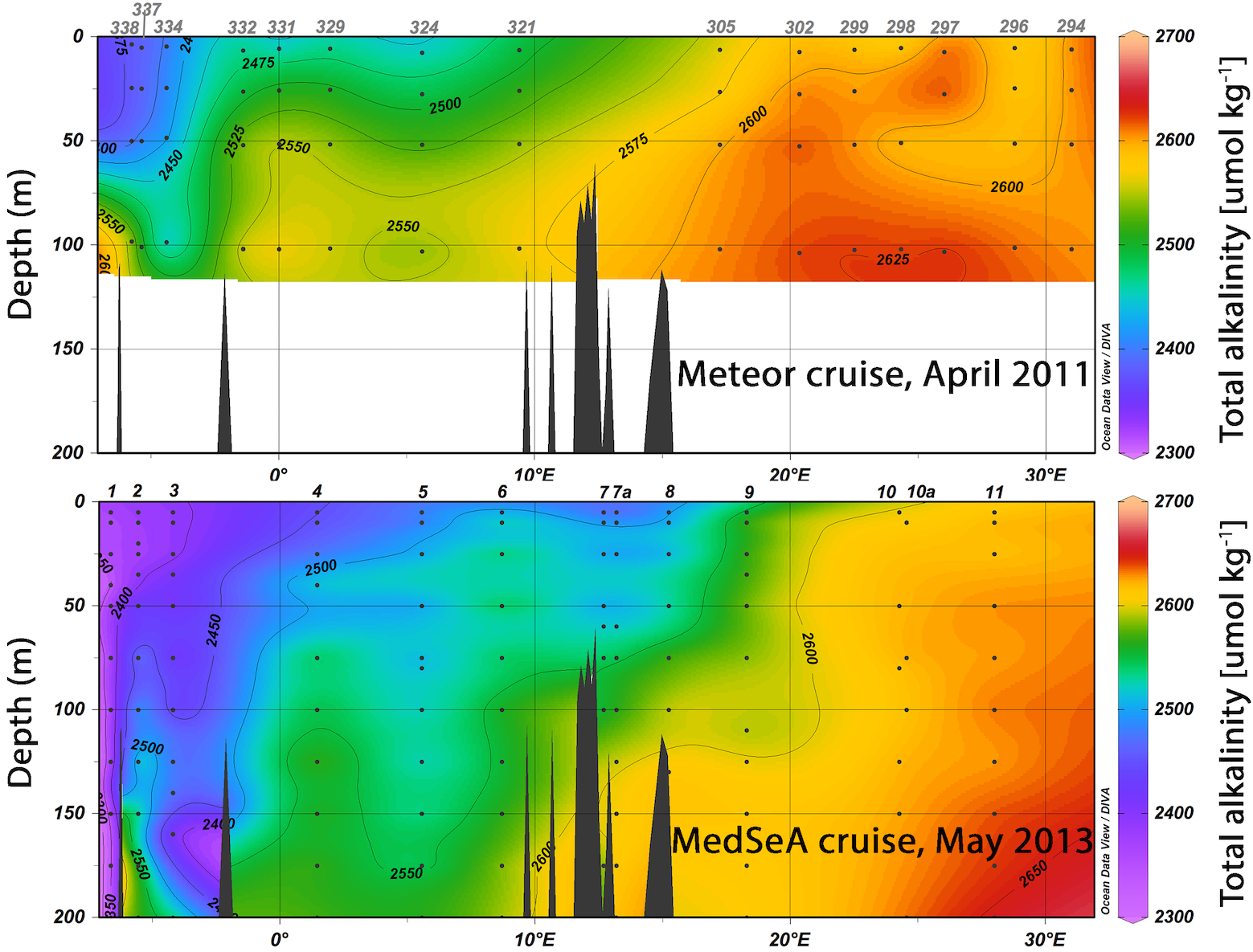

Supplement: S1 Fig — (TIF) [file pone.0201161.s001.tif]

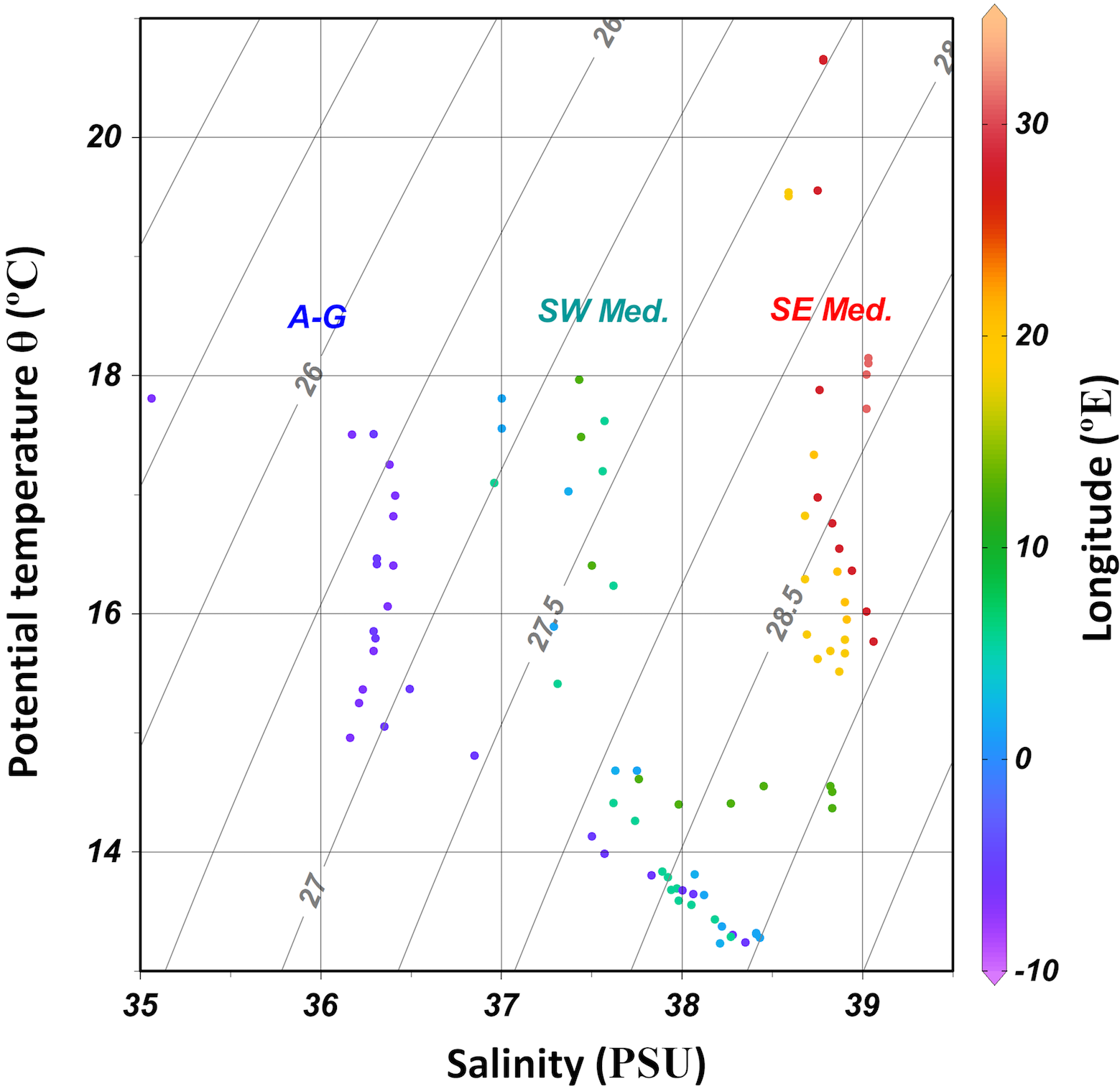

Supplement: S2 Fig — A-G = Atlantic Water from the eastern Atlantic Ocean and the Gibraltar Strait; SW Med. = Modified Atlantic Water from the South Western Mediterranean; SE Med. = Modified Atlantic Water from the South Eastern Mediterranean. (TIF) [file pone.0201161.s002.tif]

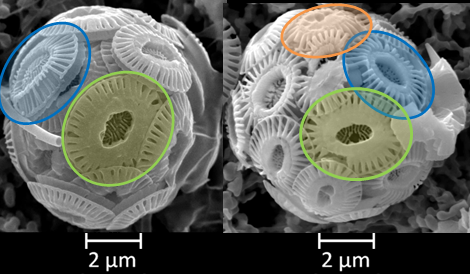

Supplement: S3 Fig — Blue = A1, green = A3b, orange = A3a. Specimens are from St. 11, collected at 75 m and 100 m depth. (TIF) [file pone.0201161.s003.tif]

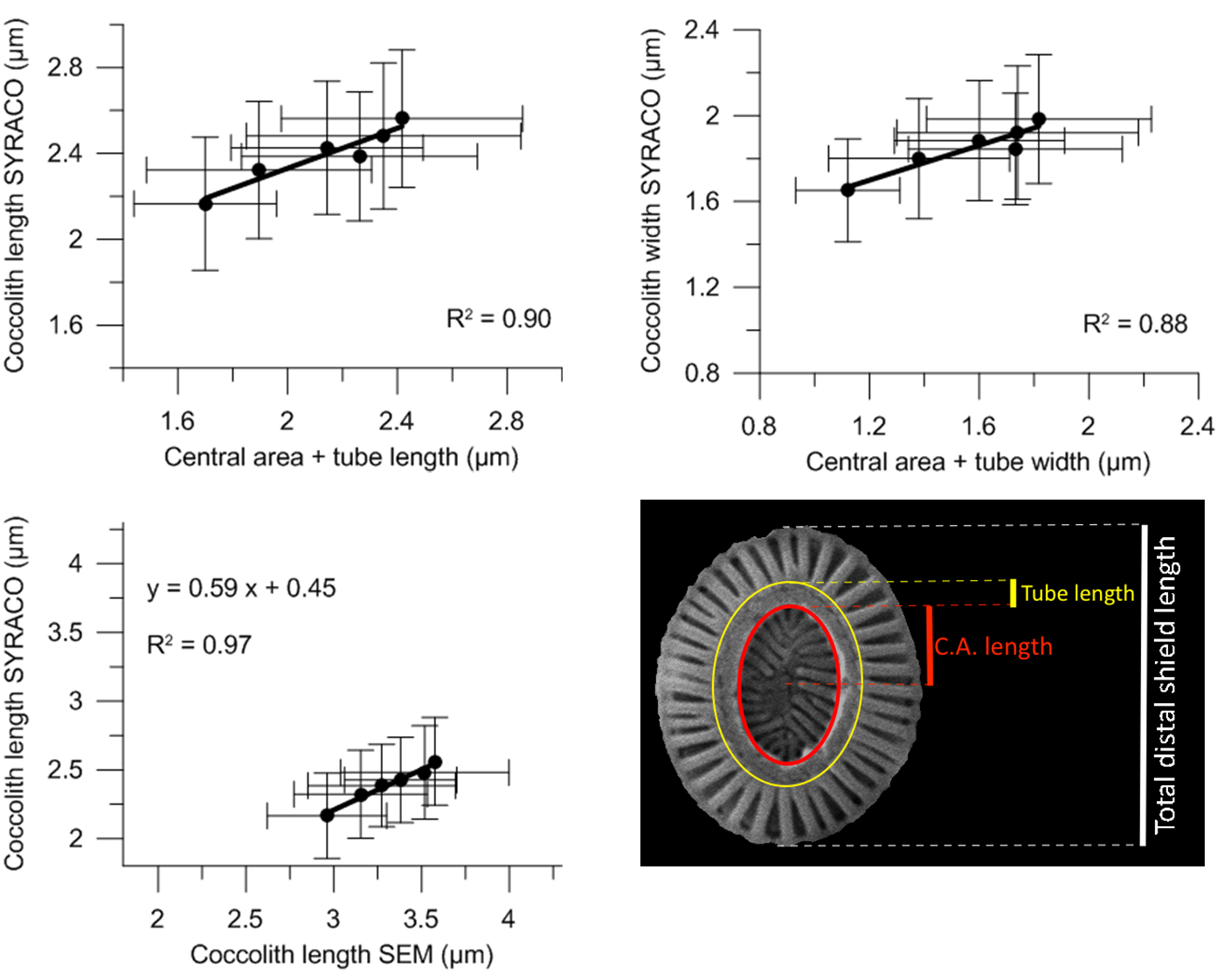

Supplement: S4 Fig — The linear regression function in the last plot was used to calculate the average corrected length (Lc) for all samples. (TIF) [file pone.0201161.s004.tif]

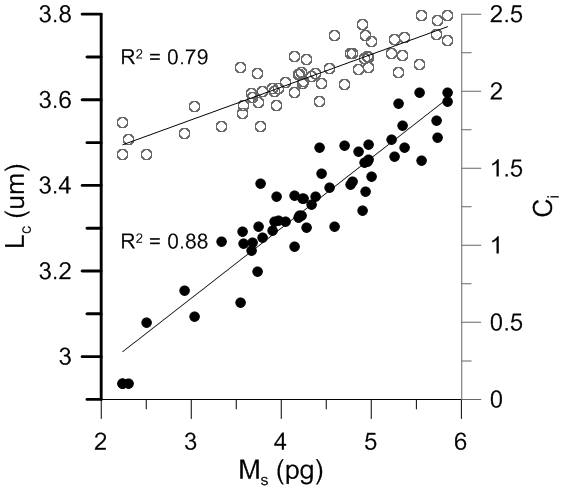

Supplement: S5 Fig — (TIF) [file pone.0201161.s005.tif]

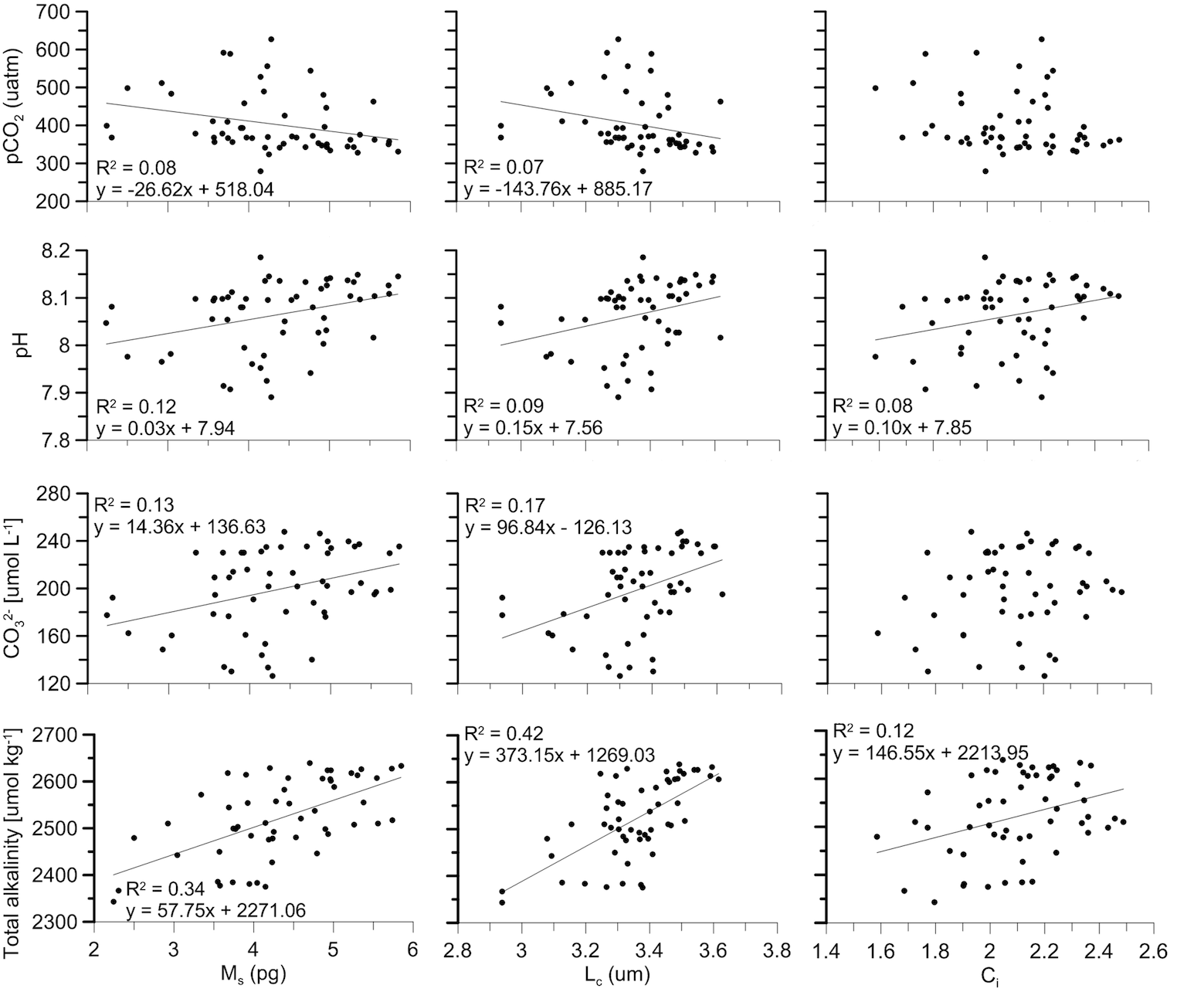

Supplement: S6 Fig — Linear regressions are shown only for combinations of parameters which have significant Spearman correlation coefficients (p ≤ 0.05). (TIF) [file pone.0201161.s006.tif]

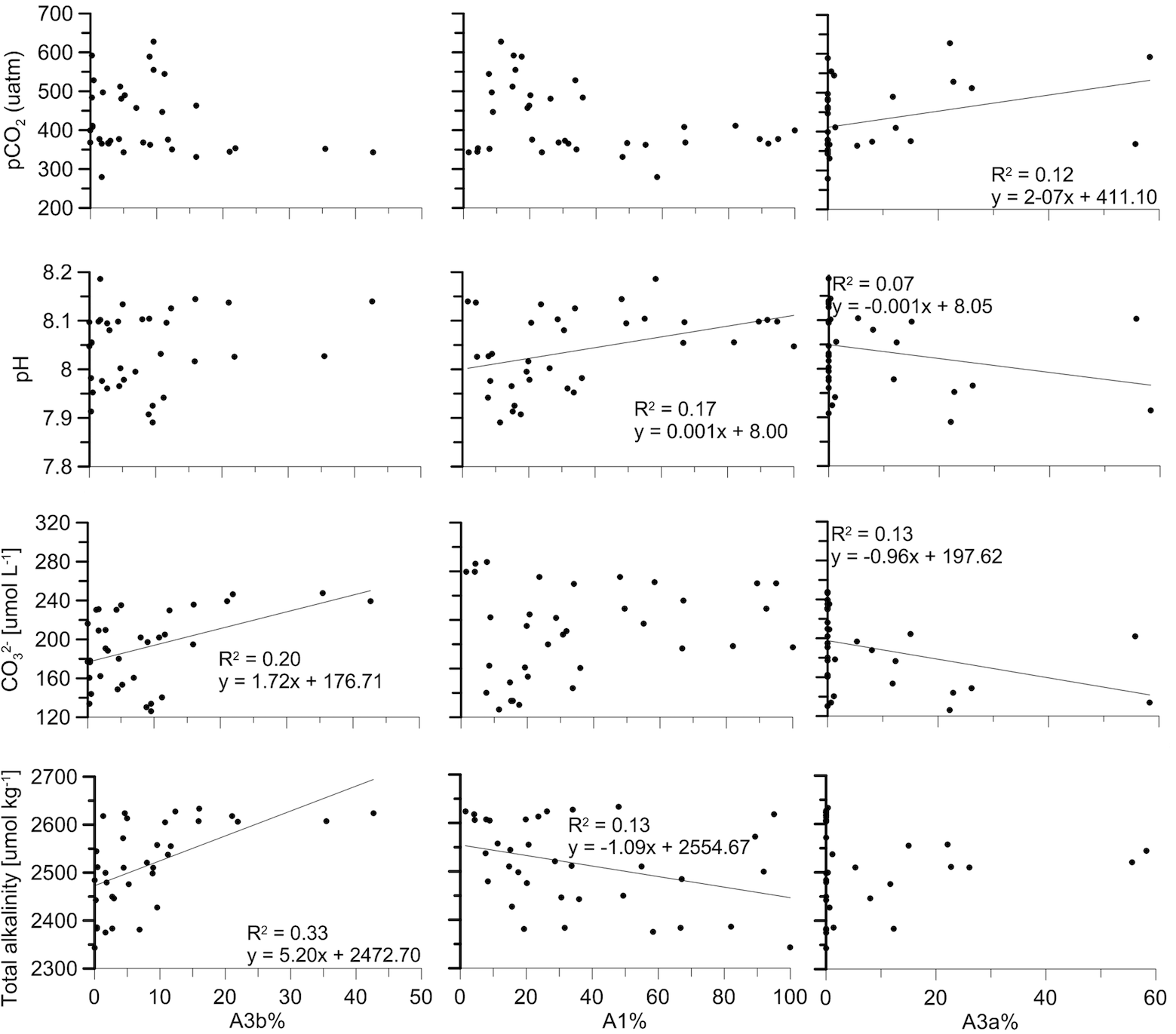

Supplement: S7 Fig — Linear regressions are shown only for combinations of parameteres which have significant Spearman correlation coefficients (p ≤ 0.05). (TIF) [file pone.0201161.s007.tif]
