# Supplementary material for: Emiliania huxleyi coccolith calcite mass modulation by morphological changes and ecology in the Mediterranean Sea
Source: PLoS One. 2018 Jul 24;13(7):e0201161. doi: 10.1371/journal.pone.0201161 (PMC6057672; doi:10.1371/journal.pone.0201161)
Supplement: S1 Table — The variety A2 is not included being very common (showing no special patterns in our samples) and medium-calcified. (DOCX) [file pone.0201161.s008.docx]

| **Calcification variety** | **Similar morphology seen in** | **Coccolith/coccosphere original description** | **Sample collection details** | | | |
| --- | --- | --- | --- | --- | --- | --- |
|  |  |  | **Sample type** | **Location** | **Season** | **Year** |
| A1 | Triantaphyllou et al., 2010 | "from high temperature water assemblage" | water | Aegean Sea (SE Med) | August-September | 2004-2008 |
|  | Beaufort, Heussner, 2001 | "open morphotype" | sediment trap | Bay of Biscay (N Atlantic) | June-September | 1990 |
| A3a | Beaufort, Heussner, 2001 | "closed morphotype" | sediment trap | Bay of Biscay (N Atlantic) | June-September | 1990 |
|  | Cros, Fortuño, 2002 | "filled central area and overcalcified appearence" | water | Balearic Islands (NW Med) | September | 1996 |
|  | Smith et al., 2012 | "overcalcified" | water | Bay of Biscay (N Atlantic) | December-March | 2008-2009 |
|  | Triantaphyllou et al., 2010 | "from low temperature water assemblage" | water | Aegean Sea (SE Med) | January-March | 2002-2008 |
| A3b | Triantaphyllou et al., 2010 | "from low temperature water assemblage" | water | Aegean Sea  (SE Med) | January-March | 2002-2008 |
|  | Dimiza et al., 2008b | "overcalcified specimen" | water | Aegean Sea  (SE Med) | April | 2002 |
|  | Dimiza et al., 2008a | - | water | Aegean Sea  (SE Med) | February | 2008 |
